# Application effect of psychological counseling intervention based on WeChat platform in chemotherapy of gastrointestinal tumors with completely implanted venous infusion ports

**DOI:** 10.1097/MD.0000000000039870

**Published:** 2024-10-11

**Authors:** Yufeng Lin, Zhiyun Cai, Lihui Lin, Yafang Ye, Bingyu Wu, Chunping Zhang

**Affiliations:** a Department of Gastrointestinal Surgery Xiamen, Zhongshan Hospital Affiliated to Xiamen University, Fujian, China; b Department of Gastroenterology Xiamen, Zhongshan Hospital Affiliated to Xiamen University, Fujian, China.

**Keywords:** adverse events, completely implanted venous infusion port, compliance, gastrointestinal tumors, psychological counseling, WeChat platform

## Abstract

This study’s objective was to observe the application effect of psychological counseling intervention based on the WeChat platform for patients with gastrointestinal tumors undergoing chemotherapy with completely implanted venous infusion ports. From October 2022 to December 2023, 110 patients with gastrointestinal tumors undergoing chemotherapy with completely implanted venous infusion ports were selected from our hospital. Patients were divided into 2 groups according to the different types of care. The control group received routine psychological counseling intervention, while the WeChat group received psychological counseling intervention based on the WeChat platform in addition to the routine intervention provided to the control group. A comparison was made between the 2 groups in terms of adverse events, compliance, hope level scores, and changes in coping strategy scores. The WeChat group experienced 7 adverse events, with an occurrence rate of 12.73%, while the control group experienced 17 adverse events, with an occurrence rate of 30.91%. The occurrence rate of adverse events in the WeChat group was significantly lower than that in the control group, showing a statistically significant difference (*P* < .05). The hope level scores before intervention in the 2 groups were compared (*P* > .05). Compared to before intervention, both groups showed an increase in scores for life attitude, proactive behavior, intimate relationships, and total scores. The WeChat group had higher scores after intervention compared to the control group (*P* < .05). The coping strategy scores before intervention in the 2 groups were compared (*P* > .05). Compared to before intervention, both groups showed an increase in facing scores and a decrease in avoidance and resignation scores (*P* < .05). After intervention, the WeChat group had higher facing scores and lower avoidance and resignation scores compared to the control group (*P* < .05). The overall chemotherapy compliance in the WeChat group was higher than that in the control group (*P* < .05). Psychological counseling intervention based on the WeChat platform can improve the level of hope, promote patients to face the disease with a positive attitude, increase chemotherapy compliance, and reduce the occurrence of adverse events in patients with gastrointestinal tumors undergoing chemotherapy with completely implanted venous infusion ports.

## 1. Introduction

In the field of gastrointestinal tumor treatment, chemotherapy is an important therapeutic method. In order to improve the efficiency of chemotherapy drug administration and the comfort of patients, completely implantable venous infusion ports have become an important auxiliary treatment device. This infusion port can provide patients with a long-term, stable intravenous infusion pathway, reducing the risk of vascular irritation and venous inflammation caused by chemotherapy drugs, and improving the continuity and safety of treatment.^[1]^ However, research has shown that during the use of completely implantable venous infusion ports, patients’ self-care abilities regarding the port and their psychological state have a significant impact on treatment outcomes. On one hand, good self-care abilities regarding the port not only reduce unnecessary physical damage to the port but also enable patients to promptly identify potential risk factors, thereby ensuring the normal standby status of the port.^[2]^ On the other hand, a positive psychological state not only enhances patients’ treatment compliance but also improves their quality of life.^[3]^ Although the current nursing model often focuses more on technical operations and basic care, with relatively little attention given to psychological care for patients. WeChat is currently the most used messaging software in China, providing users with chat, video calls, moments, and other functions. Moreover, given the popularity and convenience of WeChat in modern society, it provides a new channel for psychological support for patients. Psychological counseling interventions based on the WeChat platform can facilitate instant communication between medical staff and patients, providing personalized, continuous psychological support, and education. This helps patients better understand and manage their disease status, alleviate psychological pressure, and improve self-care abilities.^[4,5]^ Therefore, this study aims to explore the application effect of psychological counseling intervention based on the WeChat platform in the chemotherapy of gastrointestinal cancer patients with completely implanted venous infusion ports. It aims to fill the gap of psychological care in traditional nursing models and hopes to provide a new perspective and approach for the comprehensive treatment strategy of gastrointestinal cancer patients.

## 2. Materials and methods

### 2.1. General data

This study was approved by the Ethics Committee of Zhongshan Hospital Affiliated to Xiamen University (2024-0109-No.14). From October 2022 to December 2023, 110 patients with gastrointestinal tumors who underwent chemotherapy with completely implanted venous infusion ports at our hospital were selected. They were divided into 2 groups according to the different types of care. Inclusion criteria: (1) it was diagnosed as gastrointestinal tumor by pathological examination; (2) no contraindication of implantable venous access port; (3) age ≥18 years old, ≤75 years old; (4) the estimated survival time was ≥6 months, and long-term chemotherapy was required; (5) no history of long-term use of anticoagulant drugs; (6) conscious, can communicate and read normally; (7) there was no implantable infusion port before; (8) are voluntary accession, and signed the consent. Exclusion criteria: (1) ulcer or rash or infection at the puncture site; (2) complicated uncontrollable infection; (3) allergic to chemotherapy drugs; (4) there is organic damage in important organs; (5) with mental illness. The conventional group received conventional psychological counseling intervention, while the WeChat group received psychological counseling intervention based on the WeChat platform in addition to the conventional intervention received by the conventional group. A comparison of general data between the 2 groups showed comparability (*P* > .05) (see Table 1).

**Table 1:** Comparison of 2 groups of general data.

General data	Conventional group (n = 55)	WeChat group (n = 55)	*χ*^2^/t value	*P* value
Male [n, %]	34 (61.82)	31 (56.36)	0.339	.561
Female [n ,%]	21 (38.18)	24 (43.64)		
Age (years old,$\bar{X}\pm s$)	61.47 ± 4.52	60.35 ± 4.18	1.349	.180
Body mass index (kg/m^2^,$\bar{X}\pm s$)	20.14 ± 1.63	19.88 ± 1.47	0.878	.382
Tumor type [n, %]			0.582	.446
Carcinoma of stomach	26 (47.27)	30 (54.55)		
Colorectal cancer	29 (52.73)	25 (45.45)		
Academic credentials [n, %]			1.124	.570
Junior high school and below	18 (32.73)	16 (29.09)		
Secondary and high school	24 (43.64)	21 (38.18)		
College or higher	13 (23.64)	18 (32.73)		
Family average income [n, %)]			0.683	.711
<2500 yuan	24 (43.64)	20 (36.36)		
2500–5000 yuan	17 (30.91)	18 (32.73)		
More than 5000 yuan	14 (25.45)	17 (30.91)		

### 2.2. Methods

The conventional group received conventional psychological counseling intervention: (1) preliminary assessment: before the start of chemotherapy, a preliminary assessment of the patient’s mental health was conducted to understand their psychological status, including their levels of anxiety and depression, as well as their cognitive and emotional responses to the disease and treatment. (2) Provision of informational support: patients were provided with information about the treatment process, potential physical and emotional reactions they may encounter, coping strategies, etc, to help them establish realistic treatment expectations. (3) Coping strategy training: patients were taught some basic emotional regulation and coping strategies, such as relaxation training, deep breathing exercises, positive thinking, etc, to help them cope with the emotional stress during chemotherapy. (4) Port care: regularly inspect the site of the infusion port, observe for any signs of infection, leakage, or skin damage. Ensure the use of aseptic techniques when operating the port, including sterile dressing changes and aseptic handling of infusion equipment. Additionally, explain to patients and their families the function, usage, and precautions of the infusion port to ensure they understand routine care and potential complications. Educate patients to recognize signs of port-related infections, such as redness, pain, and fever.

The WeChat group adopted psychological counseling intervention based on the WeChat platform, building upon the conventional intervention received by the conventional group: (1) establishment of a WeChat platform psychological counseling team: the team consisted of 4 members, including the head nurse and responsible nurses. The team’s tasks included developing a WeChat public account psychological counseling plan, regularly assessing the intervention’s effectiveness, and collecting patient feedback to optimize services. Team members received training on how to effectively communicate and intervene using the WeChat platform, understanding its functions and limitations, and learning skills for online communication and privacy protection. (2) Creating a WeChat official account and group for psychological counseling: firstly, introduce to patients the usage process and precautions of the psychological counseling WeChat official account and group. Also, explain the types of content the WeChat official account will provide, such as mental health knowledge, disease-related information, treatment progress reminders, etc. Additionally, describe the discussion topics and activity arrangements in the WeChat group, so patients understand the assistance they can receive from it. (3) Education and information provision: regularly push graphic and textual information as well as some videos to the group, mainly covering the basic knowledge of gastrointestinal tumors, including etiology, symptoms, stages of development, and possible complications, to help patients better understand their disease status. Provide detailed explanations of the chemotherapy process, commonly used drugs, possible side effects, and their management methods, as well as the impact of chemotherapy on patients’ quality of life, allowing patients to have a clearer expectation of the treatment process. Offer guidelines on how to properly care for a venous infusion port, including key points for daily care, identifying and handling possible complications, and how to safely protect the infusion port at home. Share strategies for coping with disease-related psychological stress, anxiety, and depression, providing relaxation techniques, positive thinking training, and methods for emotional management. (4) Conducting online psychological counseling: distribute anxiety and depression assessment questionnaires^[6,7]^ within the group. For patients experiencing negative emotions, provide one-on-one online psychological counseling. Engage in deep conversations with patients, establishing trust through listening and empathy, understanding their main concerns, the sources of their anxiety, and the fluctuations in their emotions. Teach patients techniques to cope with stress and anxiety, such as deep breathing, mindfulness meditation, and emotion regulation strategies, to help them better manage their emotions in daily life. Share positive case studies from other patients to enhance their confidence and motivation. Additionally, provide patients with extra psychological health resources, such as recommended reading materials, videos, or online courses, to help them gain deeper insights into and manage their emotional issues. (5) Hosting online support groups: regularly organize online support groups within the WeChat community. Each session focuses on a specific topic, such as coping with chemotherapy side effects, dietary nutrition, emotional management, etc, encouraging patients to share their stories and experiences. Sharing participants can be notified in advance to prepare their sharing content, or an open sharing segment can be set up during the meeting, allowing willing patients to speak at any time. Encourage patients to express their emotions, providing opportunities for mutual listening and understanding, allowing patients to feel the warmth and support of the group. (6) Mental health education: regularly post-mental health tips within the WeChat group, covering diverse topics such as emotional management, stress relief, improving sleep quality, self-acceptance, mindfulness practices, and more, catering to the needs of different patients. Periodically invite mental health experts to conduct online lectures or Q&A sessions within the group, providing deeper psychological support and advice. Collect feedback and suggestions from patients on mental health education content regularly, and adjust and optimize future educational content based on this feedback.

### 2.3. Observed indexes

Adverse events such as catheter displacement, phlebitis, catheter blockage, thrombosis, infection, drug extravasation, broken tube, and skin damage were recorded in the 2 groups.

Herth hope scale^[8]^: consisting of 12 items covering 3 dimensions: life attitude, proactive behavior, and intimate relationships. A higher score indicates a higher level of hope. For coping style assessment: there are 20 subitems covering 3 coping styles: confronting, avoiding, and yielding. The coping style with the highest score suggests that the patient is likely to predominantly utilize that particular coping mechanism when facing problems.

Chemotherapy compliance: (1) full compliance: follow all medical advice without exception. (2) Partial compliance: fail to complete 1 or 2 out of the 3 tasks: medication, catheter maintenance, or rehabilitation exercise. (3) Noncompliance: fail to complete any 1 of the 3 tasks: medication, catheter maintenance, or rehabilitation exercise.

### 2.4. Statistical processing

The data were processed by SPSS26.0. It has been confirmed that the hope level score and the indwelling time of the infusion tube all obey the normal distribution, and meet the homogeneity of variance, expressed as ($\bar{X}\pm s$). The *t* test was used for comparison, and the count data such as adverse events and successful puncture were described by [n, (%)]. The rank sum test or *χ*^2^ test was used for comparison, and α = 0.05 was used as the standard.

## 3. Results

### 3.1. Comparison of 2 groups of adverse events

The WeChat group had 7 adverse events, with an incidence rate of 12.73%; the conventional group had 17 adverse events, with an incidence rate of 30.91%. The incidence rate of adverse events in the WeChat group was lower than that in the conventional group (*P* < .05) (see Table 2).

**Table 2:** Comparison of 2 groups of adverse events [n, (%)].

Adverse events	Conventional group (n = 55)	WeChat group (n = 55)	χ^2^ value	*P* value
Catheter displacement	3 (5.45%)	1 (1.82%)		
Phlebitis	0 (0.00%)	0 (0.00%)		
Catheter blockage	7 (12.73%)	4 (7.27%)		
Thrombosis	1 (1.82%)	0 (0.00%)		
Infection	2 (3.64%)	1 (1.82%)		
Medicine exosmosis	1 (1.82%)	0 (0.00%)		
Broken pipe	2 (3.64%)	1 (1.82%)		
Skin damage	1 (1.82%)	0 (0.00%)		
Total adverse events	17 (30.91%)	7 (12.73%)	5.330	.021

### 3.2. Comparison of hope level between 2 groups

The hope level scores of the 2 groups before intervention were compared (*P* > .05). Compared with before intervention, the scores of life attitude, positive actions, intimate relationships, and total scores increased in both groups, with the intervention group via WeChat showing higher improvements compared to the conventional group (*P* < .05) (see Table 3).

**Table 3:** Comparison of hope level between 2 groups (score, $\bar{X}\pm \;s$).

Group	Number of cases	Life attitude		Positive actions		Intimate relationships		Total score	
		Before intervention	After intervention	Before intervention	After intervention	Before intervention	After intervention	Before intervention	After intervention
Conventional group	55	10.11 ± 1.21	11.74 ± 1.45*	9.89 ± 1.41	11.53 ± 1.57*	10.12 ± 1.44	12.02 ± 1.51*	30.12 ± 3.29	32.29 ± 3.41*
WeChat group	55	10.04 ± 1.34	12.56 ± 1.57*	9.94 ± 1.35	12.65 ± 1.65*	10.05 ± 1.51	12.97 ± 1.77*	30.03 ± 3.47	38.18 ± 3.72*
t value		0.288	2.846	0.190	3.647	0.249	3.028	0.140	8.656
*P* value		.774	.005	.850	.000	.804	.003	.889	.000

* *P* < .05, compared with before intervention.

### 3.3. Comparison of coping style scores between the 2 groups

Before the intervention, the coping style scores of the 2 groups were compared (*P* > .05). Compared with before the intervention, the coping scores increased in both groups, while avoidance and yielding scores decreased (*P* < .05). After the intervention, the WeChat group showed higher coping scores compared to the conventional group, and lower avoidance and yielding scores compared to the conventional group (*P* < .05) (see Table 4).

**Table 4:** Comparison of coping style scores between the 2 groups (score, $\bar{X}\pm s$).

Group	Number of cases	Face		Avoidance		Yielding	
		Before intervention	After intervention	Before intervention	After intervention	Before intervention	After intervention
Conventional group	55	18.06 ± 3.41	22.44 ± 3.12*	20.12 ± 2.98	15.42 ± 2.54*	14.25 ± 2.12	10.47 ± 1.98*
WeChat group	55	17.97 ± 3.42	26.04 ± 3.75*	19.99 ± 3.24	12.74 ± 2.16*	14.09 ± 2.06	8.94 ± 1.33*
t value		0.138	5.473	0.219	5.961	0.401	4.757
*P* value		.890	.000	.827	.000	.689	.000

* *P* < .05, compared with before intervention.

### 3.4. Comparison of chemotherapy compliance between the 2 groups

Table 5 compares chemotherapy adherence between the conventional group and the WeChat group. Both groups consisted of 55 patients each. In the conventional group, 23 patients (41.82%) demonstrated full adherence, 20 patients (36.36%) showed partial adherence, and 12 patients (21.82%) were noncompliant. In contrast, the WeChat group had 34 patients (61.82%) with full adherence, 18 patients (32.73%) with partial adherence, and only 3 patients (5.45%) who were noncompliant. Statistical analysis revealed a Z value of 2.516 and a *P* value of .012, indicating a statistically significant difference in adherence between the 2 groups. The WeChat group exhibited significantly higher chemotherapy adherence compared to the conventional group.

**Table 5:** Comparison of chemotherapy compliance between the 2 groups [n, (%)].

Group	Number of cases	Full compliance	Partial compliance	Noncompliance
Conventional group	55	23 (41.82)	20 (36.36)	12 (21.82)
WeChat group	55	34 (61.82)	18 (32.73)	3 (5.45)
Z value		2.516		
*P* value		.012		

## 4. Discussion

With the advancement of medical technology, fully implantable venous access ports are widely used in the chemotherapy treatment of gastrointestinal cancer patients as a means of long-term intravenous drug administration. This device can reduce the stimulation of chemotherapy drugs on surrounding blood vessels and lower vascular-related complications, thus improving the quality of life for patients.^[9]^ However, the psychological stress faced by patients during chemotherapy cannot be overlooked, making effective psychological counseling a key factor in improving the effectiveness of chemotherapy.^[10]^ In recent years, with the rapid development of information technology, social media, especially WeChat, has become an indispensable part of people’s daily communication. Psychological counseling based on the WeChat platform not only overcomes the limitations of time and space but also provides a more convenient and interactive intervention method.^[11]^ However, despite this, there is still insufficient research on the effectiveness of psychological counseling based on the WeChat platform in the chemotherapy of gastrointestinal cancer patients with fully implantable venous access ports. This study aims to explore the application effectiveness of psychological counseling intervention based on the WeChat platform in such patients, providing a new psychological intervention approach for other long-term treatment patients.

The results of this study indicated that the indwelling time of infusion ports in the WeChat group was significantly longer than that in the conventional group, and the number of maintenance interventions was also significantly lower than that in the conventional group. This may be attributed to the fact that the WeChat-based intervention improved patients’ understanding of infusion port care knowledge and enhanced their self-care abilities. Specifically, through regular receipt and learning of infusion port care knowledge, patients’ self-care awareness and abilities were enhanced. Patients gained a better understanding of how to properly handle infusion ports, identify and prevent potential issues, directly reducing the need for professional interventions for maintenance while also prolonging the indwelling time of the infusion ports.^[12,13]^ Furthermore, WeChat, as a highly interactive communication tool, allows patients to ask questions to nursing staff at any time and receive personalized nursing advice. This bidirectional communication mode is more effective compared to traditional one-way health education, as it can provide more precise guidance based on the actual situation and needs of patients.^[14,15]^ Moreover, WeChat groups provide a platform for mutual support and experience sharing among patients. The exchange of information and experiences among patients can provide additional insights and help each other solve problems, thereby further enhancing patients’ self-care abilities through the community effect.^[16]^ Additionally, in terms of adverse event rates, the WeChat group had lower rates of adverse events compared to the conventional group, further confirming that WeChat-based psychological counseling intervention helps improve patients’ awareness of chemotherapy and infusion port management, thus effectively reducing the occurrence of adverse events.

This research further analyzed the levels of hope and coping strategies. The results indicate that after intervention, the hope level and coping scores of the WeChat group were significantly higher than those of the conventional group, while avoidance and yielding scores were significantly lower than those of the conventional group. These findings suggest that WeChat-based intervention not only enhances patients’ positive attitudes but also promotes more proactive coping strategies, helping patients face illness and treatment in a more positive and proactive manner. The reason lies in the fact that the WeChat platform provides a channel for patients to receive continuous psychological support. This immediate and continuous support helps alleviate patients’ anxiety and fear, enhances their sense of hope and positivity, thereby promoting positive coping strategies.^[17]^ Furthermore, the WeChat platform provides a convenient channel for patients to express their emotions and concerns. Through interaction with healthcare professionals and other patients, they can learn healthier ways of managing emotions, thus reducing avoidance and yielding coping strategies.^[18,19]^ Lastly, WeChat-based intervention, through education and guidance, can help patients learn how to manage their emotions and cope with the challenges of illness. This enhances patients’ self-efficacy, which is the belief in one’s ability to control and manage illness and treatment. The improvement in self-efficacy is a key factor in promoting patients’ adoption of positive coping behaviors.^[20]^ The results of this study also indicated that chemotherapy compliance in the WeChat group was significantly higher than in the conventional group. This is because the continuous psychological support and education provided through the WeChat platform enhanced patients’ understanding of and confidence in chemotherapy, thereby reducing noncompliance behaviors caused by fear and misunderstanding. This study has several limitations. First, it is a retrospective study, which may introduce selection bias in the sample. Second, the sample size is relatively small, though this does not affect the accuracy of the study. Lastly, WeChat has greater practicality in China; similar software should be used in other regions. Future plans include conducting a multi-center study to increase the sample size and follow-up duration.

In conclusion, psychological counseling intervention based on the WeChat platform can improve hope levels, promote patients’ positive attitudes towards illness, increase chemotherapy compliance, prolong the duration of indwelling catheters, and reduce the occurrence of adverse events in patients with completely implantable venous access ports for gastrointestinal tumors undergoing chemotherapy.

## Author contributions

**Conceptualization:** Yufeng Lin, Chunping Zhang.

**Data curation:** Yufeng Lin, Zhiyun Cai, Lihui Lin, Chunping Zhang.

**Formal analysis:** Yufeng Lin, Zhiyun Cai, Lihui Lin, Chunping Zhang.

**Investigation:** Zhiyun Cai, Lihui Lin, Yafang Ye, Bingyu Wu, Chunping Zhang.

**Methodology:** Zhiyun Cai, Lihui Lin, Yafang Ye, Chunping Zhang.

**Supervision:** Yafang Ye, Bingyu Wu.

**Writing – original draft:** Yufeng Lin, Chunping Zhang.

**Writing – review & editing:** Yufeng Lin, Chunping Zhang.
